# Color‐Guided Long‐Pulsed 532/1064‐nm KTP/Nd:YAG Laser for Post‐Acne Erythema: A Real‐World Study

**DOI:** 10.1111/jocd.71205

**Published:** 2026-09-28

**Authors:** Jiayi Jiang, Ying Yang, Xiaoqing Wang, Jingwen Jiang, Yan Tang, Bingrong Zhou

**Affiliations:** ^1^ Department of Dermatology Jiangsu Provincial Geriatric Hospital Nanjing China; ^2^ Department of Dermatology The First Affiliated Hospital of Nanjing Medical University Nanjing China

**Keywords:** dual‐wavelength laser, KTP laser, Nd:YAG laser, post‐acne erythema, real‐world evidence, vascular laser

## Abstract

**Purpose:**

Post‐acne erythema (PAE) is a common sequela of inflammatory acne. Pulsed dye laser is effective for vascular erythema but is associated with purpura and downtime. This study evaluated the real‐world effectiveness and tolerability of a standardized, color‐guided long‐pulsed 532/1064‐nm KTP/Nd:YAG protocol for PAE.

**Methods:**

This single‐center retrospective cohort study screened 147 patients and included 136 eligible patients. All patients completed three sessions at 4‐week intervals. Each session comprised low‐fluence full‐face 532‐nm KTP scanning followed by lesion‐targeted treatment: 532‐nm KTP for pink‐to‐red lesions and 1064‐nm Nd:YAG for violaceous lesions, with both wavelengths used when both color patterns were present. Outcomes included an adapted CAT score, erythematous area percentage, Investigator Global Assessment, satisfaction, pain, 6‐month quantitative stability, clinical worsening, and adverse events.

**Results:**

The median CAT score decreased from 5 to 1, and the median erythematous area percentage decreased from 4.50% to 1.00% (both *p* < 0.001). Median CAT improvement rates were 58.34%, 84.52%, and 87.50% in low‐, intermediate‐, and high‐severity groups, respectively; the between‐group difference was not significant (*p* = 0.10). All patients in this cohort achieved at least 25% improvement on Investigator Global Assessment. Satisfaction was 97.79%, and the median pain score was 2/10. Transient erythema resolved within 1–3 days. One patient (0.74%) developed purpura with a small blister; no pigmentary sequelae or scarring occurred. At 6 months, CAT score and erythematous area percentage remained comparable with the 1‐month posttreatment values in the overall cohort (*p* = 0.285 and *p* = 0.346, respectively) and across baseline severity subgroups (all *p* > 0.05).

**Conclusion:**

These findings suggest that a standardized three‐session, color‐guided long‐pulsed 532/1064‐nm KTP/Nd:YAG protocol may be an effective and well‐tolerated option for PAE, with exploratory 6‐month quantitative stability, warranting confirmation in prospective controlled studies.

## 1 Introduction

Post‐acne erythema (PAE) is a common and often persistent sequela of inflammatory acne vulgaris. It presents as pink‐to‐violaceous macules or patches at sites of previously inflamed lesions and may persist for months after active inflammation resolves [1]. Although nonscarring, PAE can cause substantial cosmetic concern and psychosocial distress. It frequently coexists with postinflammatory hyperpigmentation and acne scarring, further impairing quality of life and treatment satisfaction.

Persistent vasodilation, vascular remodeling, and residual inflammation contribute to PAE. Topical brimonidine and tranexamic acid may transiently reduce erythema through vascular modulation, but their effects are often limited [2]. Vascular‐targeted laser and light‐based therapies are therefore important treatment options. Pulsed dye laser (PDL), potassium titanyl phosphate (KTP) laser, neodymium‐doped yttrium aluminum garnet (Nd:YAG) laser, and intense pulsed light have been investigated for acne‐associated erythema. The 595‐nm PDL has demonstrated favorable efficacy [3], but purpura, transient pigmentary change, and downtime may reduce acceptability. Its penetration may also be insufficient for lesions with deeper vascular components.

Wavelength selection is guided by selective photothermolysis, which considers hemoglobin absorption, tissue penetration, and epidermal melanin exposure. The 532‐nm KTP wavelength is strongly absorbed by oxyhemoglobin and preferentially targets superficial dermal vessels, making it suitable for pink‐to‐red lesions [4, 5, 6, 7]. In contrast, the 1064‐nm Nd:YAG laser penetrates more deeply and has lower melanin absorption, enabling treatment of deeper vascular structures with reduced epidermal exposure [8, 9]. These complementary properties support color‐guided wavelength selection for heterogeneous PAE lesions.

Clinical studies support the 532‐nm KTP laser for superficial facial vascular lesions and the 1064‐nm Nd:YAG laser for deeper vascular targets [5, 6, 7, 8, 9, 10]. Additional evidence on dye laser use for vascular lesions and erythematous scars further supports tailored laser protocols in cosmetic dermatology [11]. However, evidence for a standardized protocol combining full‐face 532‐nm scanning with lesion‐level wavelength selection according to erythema color remains limited in PAE. Its effectiveness, tolerability, and posttreatment stability in routine practice are also insufficiently characterized. Therefore, this retrospective real‐world study evaluated a three‐session, color‐guided long‐pulsed 532/1064‐nm KTP/Nd:YAG protocol in 136 patients with PAE, assessing effectiveness, safety, patient satisfaction, and 6‐month quantitative stability.

## 2 Methods

### 2.1 Study Design

This single‐center retrospective cohort study was conducted at the Department of Dermatology, Jiangsu Provincial Geriatric Hospital. Medical records of patients with PAE treated with a long‐pulsed 532/1064‐nm KTP/Nd:YAG laser between October 2024 and October 2025 were reviewed.

### 2.2 Inclusion and Exclusion Criteria

Eligible patients were aged ≥ 18 years, had clinically diagnosed PAE of ≥ 3 months' duration, completed three treatment sessions at 4‐week intervals, and attended follow‐up at 1 and 6 months after the final session. Exclusion criteria were incomplete key follow‐up data; laser, intense pulsed light, systemic, or topical treatment likely to affect erythema within 3 months before treatment; other energy‐based procedures during the study period; severe nodulocystic acne; active cutaneous infection; keloid tendency; or concomitant dermatoses interfering with efficacy assessment. Patient selection is shown in Figure 1.

**FIGURE 1:**
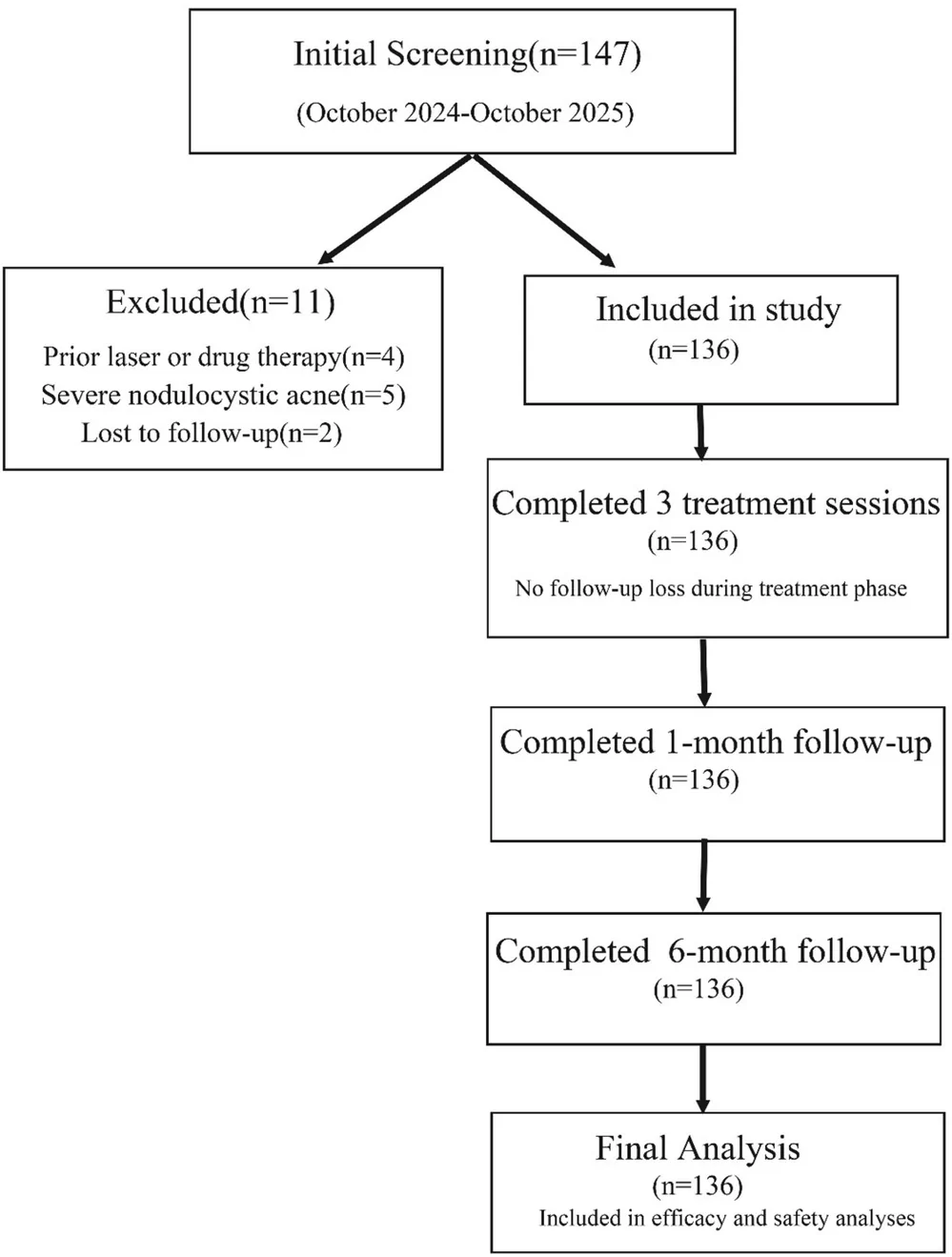
Patient selection flowchart. Of 147 patients screened, 11 were excluded for prior laser or drug therapy (*n* = 4), severe nodulocystic acne (*n* = 5), or loss to follow‐up (*n* = 2). A total of 136 patients were included in the complete‐case analysis, completed three treatment sessions, and attended both the 1‐ and 6‐month follow‐up assessments.

### 2.3 Laser Treatment Protocol

Treatments were performed using a long‐pulsed 532/1064‐nm laser system (Excel V; Cutera Inc., Brisbane, CA, USA). All patients received three sessions at 4‐week intervals, and each session followed the same two‐step treatment algorithm: standardized low‐fluence full‐face 532‐nm KTP scanning followed by color‐guided lesion‐targeted treatment.

First, the entire face was scanned with the 532‐nm KTP laser using a 10‐mm spot size, pulse duration of 10–15 ms, fluence of 3.0–4.0 J/cm^2^, repetition rate of 2 Hz, and two passes. This standardized low‐fluence step addressed diffuse erythema and concomitant mild inflammatory activity.

Second, lesion‐targeted treatment was selected according to erythema color. Pink‐to‐red lesions were treated with the 532‐nm KTP laser using a 5–7‐mm spot size, pulse duration of 10–12 ms, and fluence of 6.0–8.0 J/cm^2^. Violaceous lesions, considered clinically suggestive of relatively deeper vascular involvement, were treated with the 1064‐nm Nd:YAG laser using a 5–7‐mm spot size, pulse duration of 30–50 ms, and fluence of 50–70 J/cm^2^. Patients with both color patterns received both targeted wavelengths during the same session.

Lesion color was assessed using standardized photographs and a skin imaging system (View Skin, version 1.0.0.0; Hebei Gu'an Jingde Medical Equipment Co., Langfang, Hebei, China) by two dermatologists; a third resolved disagreements. Clinical color served as a pragmatic surrogate for presumed vascular depth. Sapphire‐window contact cooling was maintained at 5°C, and the endpoint was uniform lesion darkening without overt purpura. The same wavelength‐selection algorithm was applied at all three sessions.

### 2.4 Outcome Measures

Standardized photographs were obtained before each treatment and at 1 and 6 months after the final session. The prespecified primary efficacy assessment was 1 month after the third treatment.

The primary outcome was an adapted CAT (Clinician Erythema Assessment, Area, and Telangiectasia) score [12]. Given the absence of a validated PAE‐specific composite score, we adapted the CAT framework to capture clinically relevant dimensions (erythema intensity, area, and telangiectasia) in a single measure, and interpreted it alongside an objective imaging metric (erythematous area percentage). CAT was calculated as the Clinician Erythema Assessment (CEA) score multiplied by the facial area score, plus the telangiectasia score. CEA was graded as 0 = clear, 1 = almost clear, 2 = mild, 3 = moderate, and 4 = severe [13]. Facial involvement was estimated using the palm method: 1 = one palm area, 2 = two palm areas, and 3 = three palm areas. Telangiectasia was graded as 0 = clear, 1 = focal or mild, and 2 = diffuse or severe. CAT improvement was calculated as (pretreatment CAT—posttreatment CAT)/pretreatment CAT × 100%. Because CAT has not been formally validated for PAE, it was interpreted with the objective erythematous area percentage, defined as the proportion of total facial surface identified as erythematous by the View Skin system [14].

Secondary outcomes included Investigator Global Assessment (IGA), patient satisfaction, 6‐month quantitative stability, clinical worsening, and safety. IGA was categorized as slight or no improvement (< 25%), moderate improvement (25%–50%), marked improvement (51%–75%), or excellent improvement (>75%) [15]. Satisfaction was scored as 4 = highly satisfied, 3 = very satisfied, 2 =  satisfied, 1 = neutral, and 0 = not satisfied; overall satisfaction was the proportion of patients with scores ≥ 2 [15]. Clinical worsening was defined as an increase in CAT score or erythematous area percentage from the 1‐month assessment, confirmed by at least two independent investigators. Quantitative 6‐month stability was assessed by comparing CAT score and erythematous area percentage at 6 months with the 1‐month posttreatment values in the overall cohort and baseline severity subgroups.

For the subgroup analyses, patients were categorized into low‐severity (CEA 1), intermediate‐severity (CEA 2), and high‐severity (CEA 3–4) groups.

### 2.5 Safety Assessment

Pain during treatment was evaluated using a 10‐point visual analog scale (VAS). Adverse events were recorded, including transient erythema, persistent erythema, edema, purpura, postinflammatory hyperpigmentation, blistering, crusting, and scarring.

### 2.6 Statistical Analysis

Three dermatologists independently evaluated coded photographs while blinded to treatment parameters and other clinical data. The median of the three ratings was analyzed. Interobserver reliability of individual ratings was assessed using a two‐way mixed‐effects, absolute‐agreement, single‐measure intraclass correlation coefficient (ICC) with 95% confidence intervals.

Statistical analyses were performed using SPSS version 25.0 (IBM Corp., Armonk, NY, USA). Continuous variables are presented as medians with interquartile ranges and categorical variables as counts and percentages. Paired data were compared using the Wilcoxon signed‐rank test. Six‐month CAT and erythematous area assessments were compared with the 1‐month posttreatment values using the Wilcoxon signed‐rank test; these stability analyses were exploratory. Between‐group differences, including ordinal IGA category distributions, were assessed using the Kruskal–Wallis test, with Dunn–Bonferroni post hoc comparisons when appropriate. Other categorical variables were compared using chi‐square or Fisher exact tests. Complete‐case analysis was performed without imputation. No a priori sample‐size calculation was undertaken; all eligible patients treated during the study period were included. All tests were two‐sided, with *p* < 0.05 indicating statistical significance.

## 3 Results

### 3.1 Baseline Characteristics

Of 147 patients screened, 11 were excluded for prior laser or drug therapy (*n* = 4), severe nodulocystic acne (*n* = 5), or loss to follow‐up (*n* = 2), and 136 were included in the complete‐case analysis (Figure 1). All included patients completed three treatment sessions and the 1‐ and 6‐month follow‐up assessments. Among them, 132 (97.06%) were female and 4 (2.94%) were male. The median age was 25 years (range, 18–45 years). Fitzpatrick skin types II–III accounted for 72.79% of the cohort, whereas types IV and V accounted for 19.85% and 7.35%, respectively. At baseline, 38 patients (27.94%) had low‐severity PAE, 52 (38.24%) had intermediate‐severity PAE, and 46 (33.82%) had high‐severity PAE. Mild active acne and postinflammatory hyperpigmentation were present in 35.29% and 27.21%, respectively. Detailed characteristics are presented in Table 1.

**TABLE 1:** Baseline characteristics of included patients (*N* = 136).

Characteristic	Value
Age, years, median (range)	25 (18–45)
Sex, *n* (%)	
Male	4 (2.94)
Female	132 (97.06)
Fitzpatrick skin type, *n* (%)	
II–III	99 (72.79)
IV	27 (19.85)
V	10 (7.35)
Treatment sessions per patient, *n*	3 (all patients)
Baseline severity group, *n* (%)	
Low‐severity (CEA 1)	38 (27.94)
Intermediate‐severity (CEA 2)	52 (38.24)
High‐severity (CEA 3–4)	46 (33.82)
Mild active acne, *n* (%)	48 (35.29)
Postinflammatory hyperpigmentation, *n* (%)	37 (27.21)

*Note:* All patients received full‐face 532‐nm KTP scanning followed by color‐guided lesion‐targeted 532‐nm KTP and/or 1064‐nm Nd:YAG treatment at each of the three sessions.

Abbreviation: CEA, Clinician Erythema Assessment.

Interobserver reliability was excellent, with a single‐measure ICC of 0.93 (95% CI, 0.91–0.95).

### 3.2 Changes in CAT Score

At 1 month after the third treatment, the median CAT score decreased significantly from 5 (interquartile range [IQR], 3–8) at baseline to 1 (IQR, 0–1; *p* < 0.001). The median CAT improvement rate was 85.71% (IQR, 71.76%–100.00%).

In patients with low‐severity PAE, the median CAT score decreased from 3 (IQR, 2–3) to 1 (IQR, 0–1.25), with a median improvement rate of 58.34% (IQR, 50.00%–100.00%). In the intermediate‐severity group, the median CAT score decreased from 5 (IQR, 5–5.75) to 1 (IQR, 0–1), corresponding to an improvement rate of 84.52% (IQR, 80.00%–100.00%). In the high‐severity group, the median CAT score decreased from 10 (IQR, 8–13.25) to 1 (IQR, 0.75–2), with an improvement rate of 87.50% (IQR, 78.16%–94.65%). CAT improvement rates did not differ significantly among baseline severity groups (*p* = 0.10) (Table 2 and Figure 2).

**TABLE 2:** Changes in CAT score and erythematous area percentage after treatment according to baseline severity.

Outcome	Baseline	Posttreatment	Improvement rate (%)	Paired *p*
**CAT score**				
Overall	5 (3–8)	1 (0–1)	85.71 (71.76–100.00)	< 0.001
Low‐severity	3 (2–3)	1 (0–1.25)	58.34 (50.00–100.00)	< 0.001
Intermediate‐severity	5 (5–5.75)	1 (0–1)	84.52 (80.00–100.00)	< 0.001
High‐severity	10 (8–13.25)	1 (0.75–2)	87.50 (78.16–94.65)	< 0.001
**Erythematous area percentage (%)**				
Overall	4.50 (2.00–9.00)	1.00 (1.00–2.00)	66.67 (50.00–76.10)	< 0.001
Low‐severity	4.00 (2.00–6.00)	1.00 (1.00–2.00)	57.14 (50.00–71.25)	< 0.001
Intermediate‐severity	4.50 (3.00–8.75)	1.00 (1.00–2.00)	66.67 (50.00–79.23)	< 0.001
High‐severity	6.00 (3.00–12.25)	1.00 (1.00–2.25)	66.67 (53.63–80.19)	< 0.001

*Note:* Data are presented as median (IQR). *p* values represent paired within‐group comparisons. Between‐group *p* values for improvement rates were 0.10 for CAT and < 0.001 for erythematous area percentage. Dunn–Bonferroni testing identified a significant difference only between the low‐ and high‐severity groups for erythematous area improvement (adjusted *p* < 0.05); differences between low‐ vs. intermediate‐severity and intermediate‐ vs. high‐severity were not significant.

**FIGURE 2:**
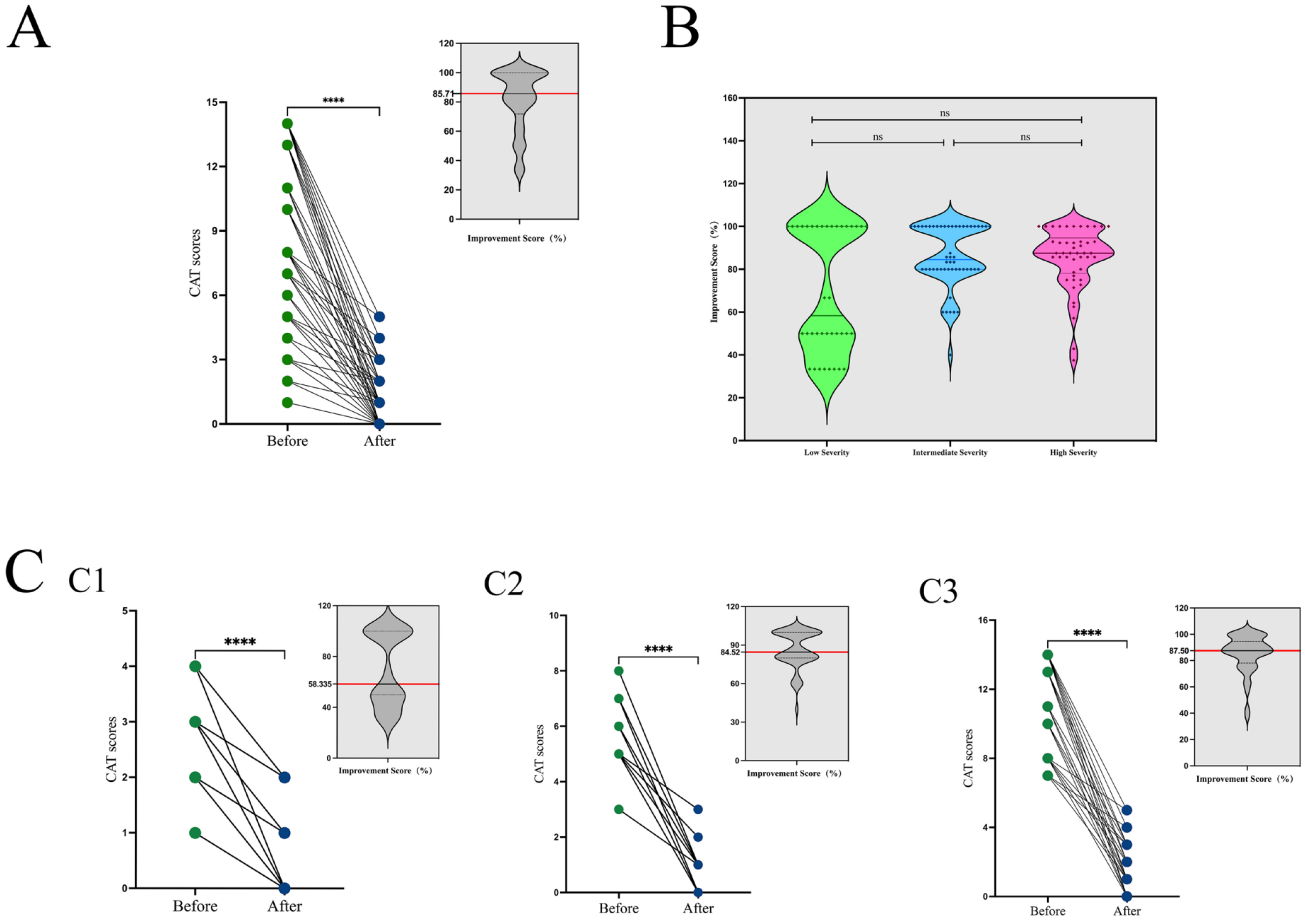
(A) CAT scores at baseline and 1 month after the third long‐pulsed KTP/Nd:YAG treatment for PAE. The median CAT score decreased from 5 (IQR, 3–8) to 1 (IQR, 0–1; *p* < 0.001). (B) Median CAT improvement rates in low‐, intermediate‐, and high‐severity groups were 58.34%, 84.52%, and 87.50%, respectively; the between‐group difference was not statistically significant (Kruskal–Wallis test, *p* = 0.10). (C) CAT scores before and after treatment according to baseline severity: (C1) low‐severity group (CEA 1, *n* = 38), from 3 (IQR, 2–3) to 1 (IQR, 0–1.25); (C2) intermediate‐severity group (CEA 2, *n* = 52), from 5 (IQR, 5–5.75) to 1 (IQR, 0–1); and (C3) high‐severity group (CEA 3–4, *n* = 46), from 10 (IQR, 8–13.25) to 1 (IQR, 0.75–2). All within‐group differences were significant (*p* < 0.001). Statistical annotations: **** indicates *p* < 0.0001, and ns indicates a nonsignificant comparison. In the inset violin plots in panels A and C1‐C3, the red horizontal line marks the median CAT improvement rate.

### 3.3 Objective Erythema Assessment

The median erythematous area percentage decreased significantly from 4.50% (IQR, 2.00%–9.00%) at baseline to 1.00% (IQR, 1.00%–2.00%) at 1 month after the third treatment (*p* < 0.001). The median improvement rate for the overall cohort was 66.67% (IQR, 50.00%–76.10%).

Median erythematous area improvement rates were 57.14% (IQR, 50.00%–71.25%), 66.67% (IQR, 50.00%–79.23%), and 66.67% (IQR, 53.63%–80.19%) in the low‐, intermediate‐, and high‐severity groups, respectively. The overall difference among groups was statistically significant (*p* < 0.001). Dunn–Bonferroni post hoc analysis identified a significant difference between the low‐ and high‐severity groups (adjusted *p* < 0.05), whereas the low‐ versus intermediate‐severity and intermediate‐ versus high‐severity comparisons were not significant (Table 2).

### 3.4 Investigator Global Assessment

At 1 month after the third treatment, all 136 patients achieved at least moderate improvement (≥ 25%) on IGA. The ordinal distribution of IGA categories did not differ significantly among baseline severity groups (*p* = 0.345; Table 3). Representative clinical photographs are shown in Figure 3.

**TABLE 3:** Investigator Global Assessment at 1 month after the third treatment according to baseline severity.

Baseline severity	< 25%	25%–50%	51%–75%	> 75%	*p*
Low‐severity (*n* = 38)	0 (0.0)	11 (28.9)	9 (23.7)	18 (47.4)	0.345
Intermediate‐severity (*n* = 52)	0 (0.0)	9 (17.3)	17 (32.7)	26 (50.0)	
High‐severity (*n* = 46)	0 (0.0)	6 (13.0)	13 (28.3)	27 (58.7)	

*Note:* Data are *n* (%). IGA categories were slight or no (< 25%), moderate (25%–50%), marked (51%–75%), and excellent (> 75%) improvement. The *p* value compares ordinal distributions using the Kruskal–Wallis test.

Abbreviation: IGA, Investigator Global Assessment.

**FIGURE 3:**
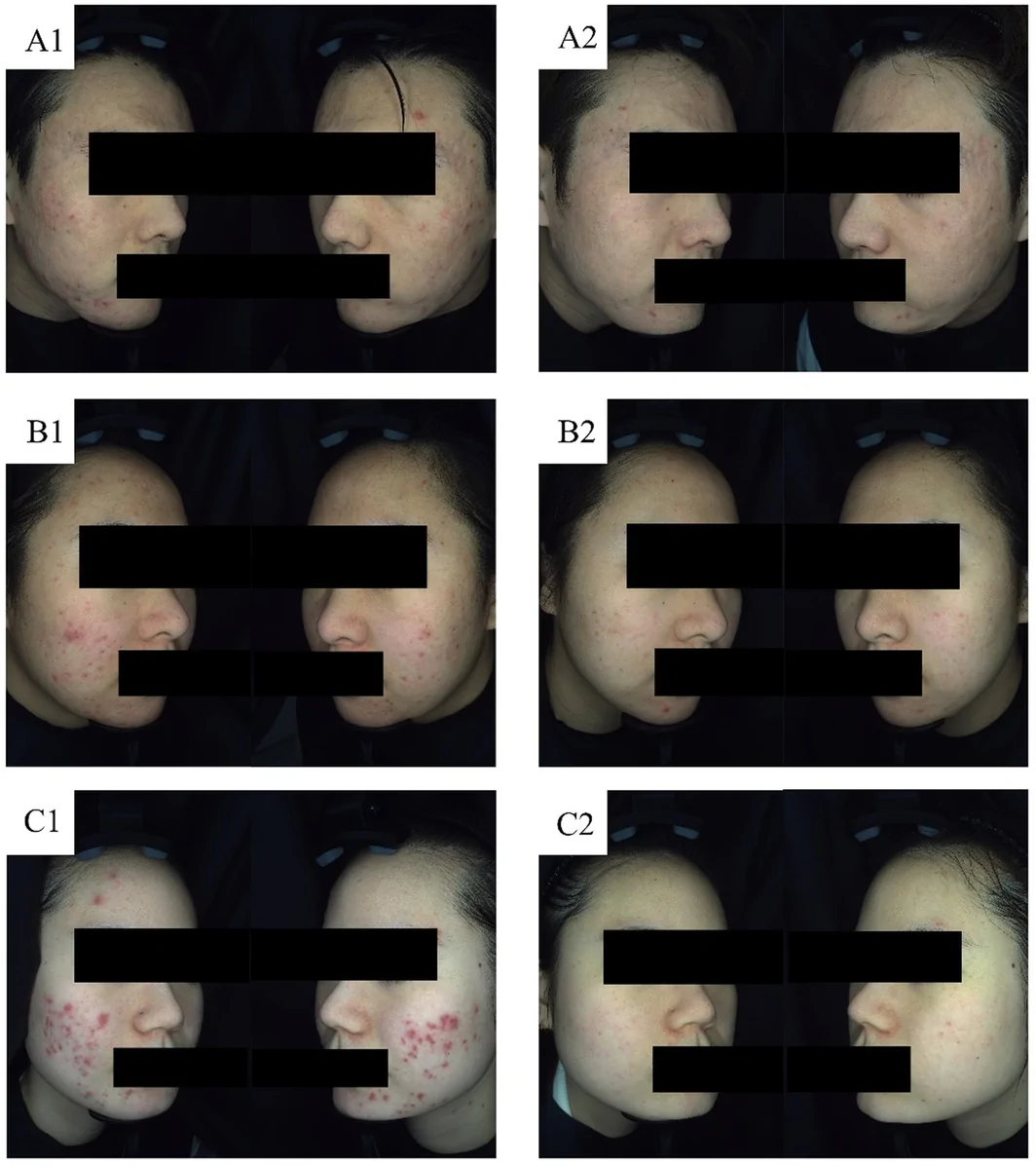
Representative standardized clinical photographs obtained at baseline and 1 month after the third long‐pulsed KTP/Nd:YAG treatment. (A) Low‐severity PAE before treatment (A1) and after three sessions (A2). (B) Intermediate‐severity PAE before treatment (B1) and after three sessions (B2). (C) High‐severity PAE before treatment (C1) and after three sessions (C2).

### 3.5 Safety, Satisfaction, and Follow‐Up

The median pain visual analog scale score was 2 (IQR, 1–3). Transient erythema was observed in 121 patients (88.97%) and resolved spontaneously within 1–3 days. One patient (0.74%) developed immediate purpura followed by a small blister 12 h later; both resolved without treatment‐emergent postinflammatory hyperpigmentation or scarring. No persistent erythema, edema, infection, crusting, or scarring was observed.

At the 6‐month follow‐up, 133 patients (97.79%) reported satisfaction scores ≥ 2, and 3 (2.21%) rated the outcome as neutral. Quantitative follow‐up showed no significant change from 1 month to 6 months in CAT score [1 (0–1) vs. 1 (0–1), *p* = 0.285] or erythematous area percentage [1.00% (1.00–2.00) vs. 1.00% (1.00–2.00), *p* = 0.346] in the overall cohort. Similar findings were observed across low‐, intermediate‐, and high‐severity groups for CAT score (*p* = 0.206, 0.564, and 0.317, respectively) and erythematous area percentage (*p* = 0.102, 0.480, and 0.317, respectively) (Table 4).

**TABLE 4:** Six‐month stability of CAT score and erythematous area percentage.

Outcome	1 month	6 months	*p*
**CAT score**			
Overall	1 (0–1)	1 (0–1)	0.285
Low‐severity	1 (0–1.25)	1 (0–2)	0.206
Intermediate‐severity	1 (0–1)	1 (0–1)	0.564
High‐severity	1 (0.75–2)	1 (0.75–2)	0.317
**Erythematous area percentage (%)**			
Overall	1.00 (1.00–2.00)	1.00 (1.00–2.00)	0.346
Low‐severity	1.00 (1.00–2.00)	1.00 (1.00–2.00)	0.102
Intermediate‐severity	1.00 (1.00–2.00)	1.00 (1.00–2.00)	0.480
High‐severity	1.00 (1.00–2.25)	1.00 (1.00–3.00)	0.317

*Note:* Data are presented as median (IQR). *p* values compare 6‐month posttreatment values with 1‐month posttreatment values using the Wilcoxon signed‐rank test.

Abbreviation: CAT, Clinician Erythema Assessment, Area, and Telangiectasia.

## 4 Discussion

This single‐center retrospective real‐world study of 136 patients with PAE found that a standardized three‐session, color‐guided long‐pulsed 532‐nm KTP/1064‐nm Nd:YAG protocol was associated with significant improvement in clinician‐assessed and instrument‐based outcomes, low procedural pain, limited downtime, and high patient satisfaction. The cohort included varied baseline severities, Fitzpatrick phototypes, and concomitant mild acne, reflecting routine clinical heterogeneity. Treatment exposure was uniform, with all patients completing three sessions at 4‐week intervals. Although the uncontrolled design precludes causal inference and spontaneous improvement cannot be excluded, concordant changes across complementary outcomes support the protocol's clinical feasibility. Exploratory quantitative follow‐up further showed that CAT score and erythematous area percentage remained statistically comparable between the 1‐ and 6‐month assessments in the overall cohort and baseline severity subgroups.

Persistent vasodilation and vascular remodeling after inflammation contribute to PAE [16], supporting vascular‐targeted treatment. The 532‐nm KTP wavelength is strongly absorbed by oxyhemoglobin and is suited to superficial dilated vessels [4, 5, 6, 7]. KTP treatment may provide improvement comparable to PDL for superficial facial vascular lesions, although prolonged posttreatment erythema has been reported [5]. In our cohort, conservative fluence, longer pulse durations, a nonpurpuric endpoint, and sapphire‐window contact cooling were associated with a median pain score of 2/10. Posttreatment erythema resolved within 1–3 days, and no persistent edema occurred, indicating limited recovery time.

The 1064‐nm Nd:YAG laser provides greater dermal penetration and lower epidermal melanin absorption than 532‐nm treatment [8, 17, 18, 19]. These properties may benefit violaceous lesions with presumed deeper vascular involvement and patients with darker phototypes. Previous studies reported erythema improvement comparable to 595‐nm PDL, with fewer adverse events [9]. In our cohort, 27.21% of patients had Fitzpatrick skin types IV–V, and no treatment‐emergent persistent postinflammatory hyperpigmentation occurred. This finding is consistent with the reported tolerability of long‐pulsed 1064‐nm treatment [20]. However, safety was not analyzed separately by phototype or targeted wavelength, precluding comparative conclusions.

The protocol was defined by a standardized two‐step structure. All patients received two low‐fluence full‐face 532‐nm scanning passes, followed by color‐guided lesion treatment: 532 nm for pink‐to‐red lesions and 1064 nm for violaceous lesions. Patients with mixed color patterns received both targeted wavelengths during the same session. This approach is clinically relevant because PAE may contain heterogeneous vascular patterns within the same face, making a fixed single‐wavelength protocol less adaptable to routine practice. The full‐face passes were intended to address diffuse erythema and concomitant mild inflammatory activity [21, 22, 23], although acne‐specific outcomes were not assessed. Vascular depth was estimated from clinical color rather than confirmed by vascular imaging. Wavelength‐specific exposures and outcomes were not analyzed separately. Therefore, the relative contributions of the full‐face and targeted components cannot be distinguished.

CAT improvement did not differ significantly among baseline severity groups, whereas erythematous area improvement differed between the low‐ and high‐severity groups. This discrepancy may reflect the dimensions captured by each measure. CAT integrates erythema intensity, lesion extent, and telangiectatic change, whereas erythematous area percentage quantifies only the proportion of facial surface classified as erythematous. Floor effects in low‐severity disease may also have limited measurable relative change. Because CAT was developed for erythematotelangiectatic rosacea rather than PAE, its use should be considered an adapted multidimensional assessment and interpreted with the objective imaging outcome.

Transient erythema was the most frequent adverse event and resolved within several days. Only one patient developed purpura followed by a small blister; both resolved without pigmentary sequelae or scarring. Previous PDL studies in PAE or erythematous acne scars reported higher purpura rates [24]. Such indirect comparisons require caution because patient characteristics, treatment parameters, endpoints, and adverse‐event definitions differ across studies. The low purpura rate may relate to conservative fluence, longer pulse durations, a nonpurpuric endpoint, and contact cooling [10, 25, 26], but these explanations cannot be confirmed without a comparative design. The study's real‐world value lies in showing that a prespecified color‐guided algorithm can be implemented across a heterogeneous cohort with low procedural discomfort and limited downtime.

Several limitations should be acknowledged. First, the absence of an untreated, single‐wavelength, or PDL comparator prevents causal attribution and direct efficacy or safety comparisons. Second, the retrospective single‐center design and exclusion of patients with incomplete follow‐up may have introduced selection and attrition bias; the predominance of women further limits generalizability. Third, clinical color was a pragmatic surrogate for vascular depth, and wavelength‐specific exposures and outcomes were not analyzed separately. Fourth, the adapted CAT score has not been validated for PAE, and active acne may have influenced erythema measurements; no multivariable adjustment was performed for phototype or postinflammatory hyperpigmentation. Finally, no a priori sample‐size calculation was performed, and follow‐up was limited to 6 months. Although CAT and erythematous area were repeated at 6 months, this stability analysis was exploratory and lacked a comparator or longer‐term data. Nevertheless, the uniform schedule, standardized assessments, blinded photographic evaluation, excellent interobserver reliability, objective imaging, and consistent within‐patient changes strengthen the internal coherence of the findings.

## 5 Conclusion

Overall, this study provides real‐world evidence that a standardized three‐session protocol combining full‐face low‐fluence 532‐nm KTP scanning with color‐guided lesion‐targeted 532‐nm KTP and/or 1064‐nm Nd:YAG treatment was associated with improvement in PAE, low procedural pain, limited downtime, and favorable tolerability. Exploratory quantitative follow‐up showed no statistically significant change in CAT score or erythematous area between 1 and 6 months after treatment. The strategy may support individualized wavelength selection when lesions of different colors coexist; however, its comparative value over single‐wavelength treatment or PDL remains uncertain. Prospective multicenter controlled studies incorporating predefined wavelength‐allocation criteria, objective vascular imaging, validated patient‐reported outcomes, and longer follow‐up are warranted.

## Author Contributions

Jiayi Jiang and Bingrong Zhou conceived the study, and all authors contributed to its design. Jiayi Jiang and Xiaoqing Wang performed the treatments. Yan Tang and Jingwen Jiang coordinated patient enrollment, questionnaires, and follow‐up. Jiayi Jiang, Ying Yang, and Xiaoqing Wang performed the photographic assessments and statistical analyses. Jiayi Jiang drafted the manuscript; Ying Yang and Bingrong Zhou revised it. All authors approved the final version.

## Funding

This work was supported by the National Natural Science Foundation of China (grant no. 82073472); the Priority Academic Program Development of Jiangsu Higher Education Institutions; the Nantong Young Medical Expert Training Plan; and the Outstanding Young and Middle‐Aged Talents Support Program of the First Affiliated Hospital of Nanjing Medical University (Jiangsu Province Hospital).

## Ethics Statement

This study was conducted in accordance with the Declaration of Helsinki and approved by the Ethics Committee of Jiangsu Provincial Geriatric Hospital (approval no. 0921‐1). Written informed consent was obtained from all participants.

## Consent

Written informed consent was obtained from all study participants.

## Conflicts of Interest

The authors declare no conflicts of interest.

## Data Availability

The data supporting the findings of this study are available from the corresponding author upon reasonable request.

## References

[1] N. Altunisik , D. Turkmen , I. Durmaz , and S. Sener , “Treatment of Post‐Acne Erythema With 585 Nm Q‐Switched Nd:YAG Laser in a Patient Receiving Isotretinoin Therapy,” Journal of Cosmetic Dermatology 21, no. 10 (2022): 5241–5243, 10.1111/jocd.14824. 35113473

[2] Y. Kalantari , S. Dadkhahfar , and I. Etesami , “Post‐Acne Erythema Treatment: A Systematic Review of the Literature,” Journal of Cosmetic Dermatology 21, no. 4 (2022): 1379–1392, 10.1111/jocd.14804. 35076997

[3] H. J. Yoon , D. H. Lee , S. O. Kim , K. C. Park , and S. W. Youn , “Acne Erythema Improvement by Long‐Pulsed 595‐Nm Pulsed‐Dye Laser Treatment: A Pilot Study,” Journal of Dermatological Treatment 19, no. 1 (2008): 38–44, 10.1080/09546630701646164. 18273723

[4] M. Adamič , M. D. Pavlović , A. Troilius Rubin , M. Palmetun‐Ekbäck , and P. Boixeda , “Guidelines of Care for Vascular Lasers and Intense Pulse Light Sources From the European Society for Laser Dermatology,” Journal of the European Academy of Dermatology and Venereology 29, no. 9 (2015): 1661–1678, 10.1111/jdv.13177. 25931003

[5] N. S. Uebelhoer , M. A. Bogle , B. Stewart , K. A. Arndt , and J. S. Dover , “A Split‐Face Comparison Study of Pulsed 532‐Nm KTP Laser and 595‐Nm Pulsed Dye Laser in the Treatment of Facial Telangiectasias and Diffuse Telangiectatic Facial Erythema,” Dermatologic Surgery 33, no. 4 (2007): 441–448, 10.1111/j.1524-4725.2007.33091.x. 17430378

[6] G. L. Becher , H. Cameron , and H. Moseley , “Treatment of Superficial Vascular Lesions With the KTP 532‐nm Laser: Experience With 647 Patients,” Lasers in Medical Science 29, no. 1 (2014): 267–271, 10.1007/s10103-013-1330-5. 24401943

[7] C. Clark , H. Cameron , H. Moseley , J. Ferguson , and S. H. Ibbotson , “Treatment of Superficial Cutaneous Vascular Lesions: Experience With the KTP 532 Nm Laser,” Lasers in Medical Science 19, no. 1 (2004): 1–5, 10.1007/s10103-004-0294-x. 15316851

[8] D. Groot , J. Rao , P. Johnston , and T. Nakatsui , “Algorithm for Using a Long‐Pulsed Nd:YAG Laser in the Treatment of Deep Cutaneous Vascular Lesions,” Dermatologic Surgery 29, no. 1 (2003): 35–42, 10.1046/j.1524-4725.2003.29016.x. 12534510

[9] N. Chalermsuwiwattanakan , S. Rojhirunsakool , N. Kamanamool , S. Kanokrungsee , and M. Udompataikul , “The Comparative Study of Efficacy Between 1064‐Nm Long‐Pulsed Nd:YAG Laser and 595‐nm Pulsed Dye Laser for the Treatment of Acne Vulgaris,” Journal of Cosmetic Dermatology 20, no. 7 (2021): 2108–2115, 10.1111/jocd.13832. 33226176

[10] A. Miller , “Treatment of Erythematotelangiectactic Rosacea With a KTP YAG Laser,” Journal of Drugs in Dermatology 4, no. 6 (2005): 760–762. 16302564

[11] A. Clementi , G. Cannarozzo , S. Amato , et al., “Dye Laser Applications in Cosmetic Dermatology: Efficacy and Safety in Treating Vascular Lesions and Scars,” Cosmetics 11, no. 6 (2024): 227, 10.3390/cosmetics11060227.

[12] J. K. Seo , E. J. Shin , K. H. Jeong , and M. K. Shin , “Clinician Severity Assessment Grading Scale on Erythematotelangiectatic Rosacea,” Indian Journal of Dermatology 66, no. 2 (2021): 203–205, 10.4103/ijd.IJD_611_19. PMC8208259 34188282

[13] J. Tan , H. Liu , J. J. Leyden , and M. J. Leoni , “Reliability of Clinician Erythema Assessment Grading Scale,” Journal of the American Academy of Dermatology 71, no. 4 (2014): 760–763, 10.1016/j.jaad.2014.05.044. 24999270

[14] L. Yang , Y. Gao , H. Wu , Y. Li , C. Li , and X. Li , “Efficacy and Safety of Intradermal Botulinum Toxin A for Post‐Acne Erythema: A Split‐Face Randomized Controlled Trial,” Frontiers in Medicine 12 (2025): 1610125, 10.3389/fmed.2025.1610125. PMC12665604 41333784

[15] L. Al‐Quran , G. Li , Z. Liu , D. Xiong , X. Cao , and T. Xie , “Comparative Efficacy Between Intense Pulsed Light Narrow Spectrum and Broad Spectrum in the Treatment of Post‐Acne Erythema (PAE),” Clinical, Cosmetic and Investigational Dermatology 16 (2023): 1983–1996, 10.2147/ccid.S419743. PMC10404044 37547541

[16] S. Min , S. Y. Park , J. Y. Yoon , H. H. Kwon , and D. H. Suh , “Fractional Microneedling Radiofrequency Treatment for Acne‐Related Post‐Inflammatory Erythema,” Acta Dermato‐Venereologica 96, no. 1 (2016): 87–91, 10.2340/00015555-2164. 26059315

[17] X. Jia , L. Zheng , L. Fang , et al., “Evaluation of the Safety and Efficacy of a Fractional Picosecond 1064 nm Laser for Post‐Acne Erythema in Adult Chinese Patients,” Skin Research and Technology 29, no. 1 (2023): e13274, 10.1111/srt.13274. PMC10155796 36704883

[18] J. Loyal , E. Carr , R. Almukhtar , and M. P. Goldman , “Updates and Best Practices in the Management of Facial Erythema,” Clinical, Cosmetic and Investigational Dermatology 14 (2021): 601–614, 10.2147/ccid.S267203. PMC8197440 34135612

[19] R. Amiri , M. Khalili , S. Mohammadi , B. Iranmanesh , and M. Aflatoonian , “Treatment Protocols and Efficacy of Light and Laser Treatments in Post‐Acne Erythema,” Journal of Cosmetic Dermatology 21, no. 2 (2022): 648–656, 10.1111/jocd.14729. 34985175

[20] H. H. M. Eltanany , F. Qawy , M. Hamdino , and M. S. Ali , “A Split Face Study Comparing Intralesional Tranexamic Acid Versus 1064 nm Long Pulsed Nd: YAG Laser for Acne Vulgaris Treatment,” Archives of Dermatological Research 317, no. 1 (2025): 709, 10.1007/s00403-025-04164-4. 40221536

[21] O. Yilmaz , N. Senturk , E. P. Yuksel , et al., “Evaluation of 532‐nm KTP Laser Treatment Efficacy on Acne Vulgaris With Once and Twice Weekly Applications,” Journal of Cosmetic and Laser Therapy 13, no. 6 (2011): 303–307, 10.3109/14764172.2011.630087. 21981360

[22] N. Sadick , “An Open‐Label, Split‐Face Study Comparing the Safety and Efficacy of Levulan Kerastick (Aminolevulonic Acid) Plus a 532 nm KTP Laser to a 532 Nm KTP Laser Alone for the Treatment of Moderate Facial Acne,” Journal of Drugs in Dermatology 9, no. 3 (2010): 229–233. 20232583

[23] W. P. Baugh and W. D. Kucaba , “Nonablative Phototherapy for Acne Vulgaris Using the KTP 532 nm Laser,” Dermatologic Surgery 31, no. 10 (2005): 1290–1296, 10.1111/j.1524-4725.2005.31205. 16188181

[24] C. Zhou , M. Yao , W. Chen , et al., “Treatment of Erythematous Acne Scars Using 595‐nm Pulsed Dye Laser Combined With 1565‐nm ResurFX Nonablative Fractional Laser,” Journal of Cosmetic Dermatology 23, no. 6 (2024): 2015–2021, 10.1111/jocd.16235. 38426374

[25] C. H. Nam , M. H. Kim , S. P. Hong , and B. C. Park , “Fractional 532‐nm KTP Diode Laser and 595‐nm Pulsed Dye Laser in Treatment of Facial Telangiectatic Erythema,” Journal of Cosmetic Dermatology 18, no. 3 (2019): 783–787, 10.1111/jocd.12692. 29974643

[26] E. F. Bernstein , A. Noyaner‐Turley , and B. Renton , “Treatment of Spider Veins of the Lower Extremity With a Novel 532 nm KTP Laser,” Lasers in Surgery and Medicine 46, no. 2 (2014): 81–88, 10.1002/lsm.22178. 24127163

